# Supplementary material for: Redefining Hypo- and Hyper-Responding Phenotypes of CFTR Mutants for Understanding and Therapy
Source: Int J Mol Sci. 2022 Dec 2;23(23):15170. doi: 10.3390/ijms232315170 (PMC9735543; doi:10.3390/ijms232315170)
Supplement: Supplementary file 1 [file ijms-23-15170-s001.zip › ijms-1938702-supplementary.pdf]

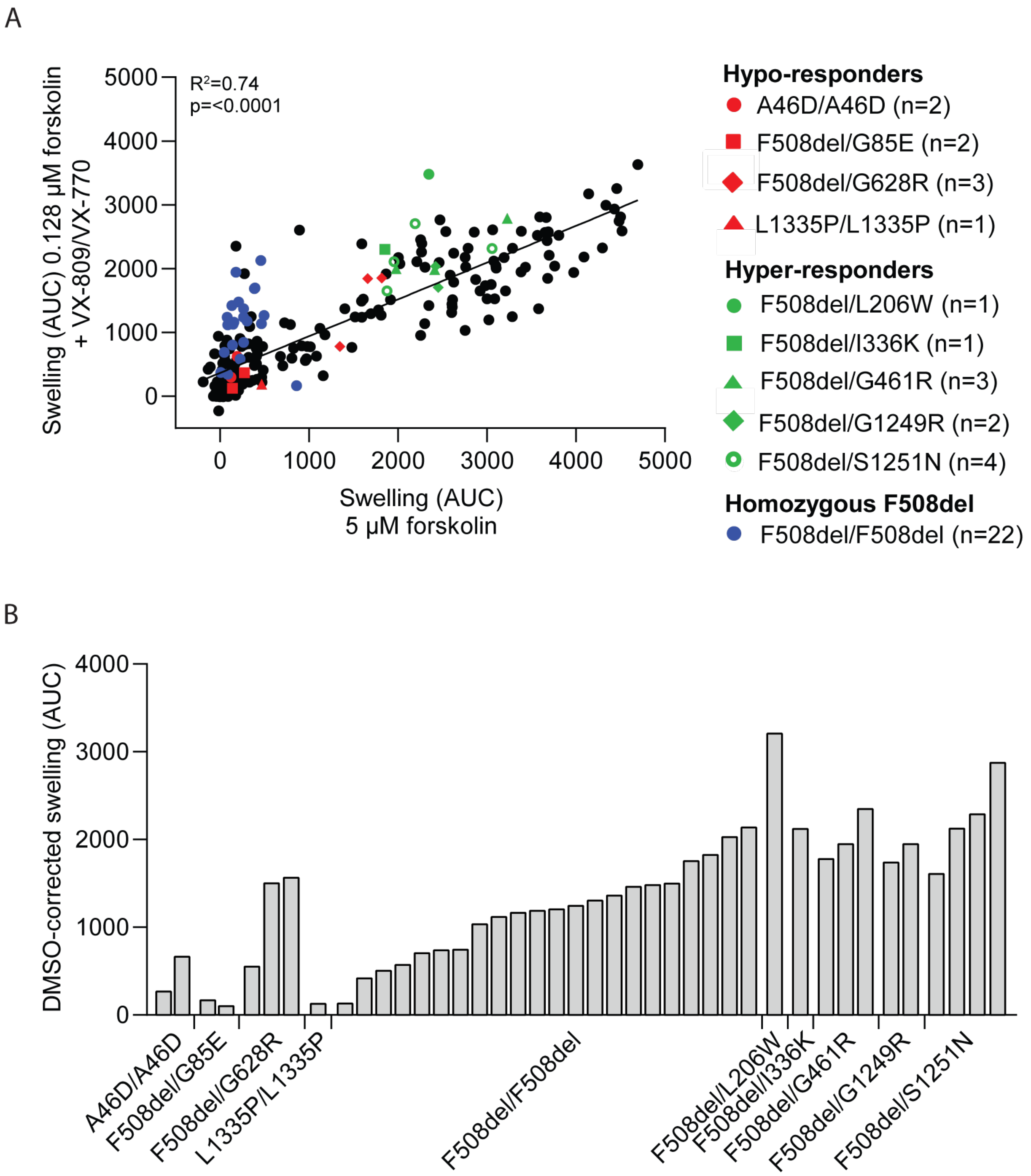

Supplementary Figure S1: Functional analysis of patient-derived intestinal organoids
(A) Correlation plot between residual function swelling (5 µM forskolin) and VX-809/VX-770-induced swelling for each patient-derived intestinal organoid (PDIO). Hypo-responders are indicated in red, hyper-responders are indicated in green and homozygous F508del PDIOs are indicated in blue. **(B)** Forskolin-(0.128 µM)-induced organoid swelling for a panel of VX-809/VX-770-treated PDIO, divided over 10 genotypes (25, 71). Swelling is quantified as AUC and corrected for DMSO-induced swelling. Averages are shown of >3 biological replicate experiments with three technical replicates per condition.

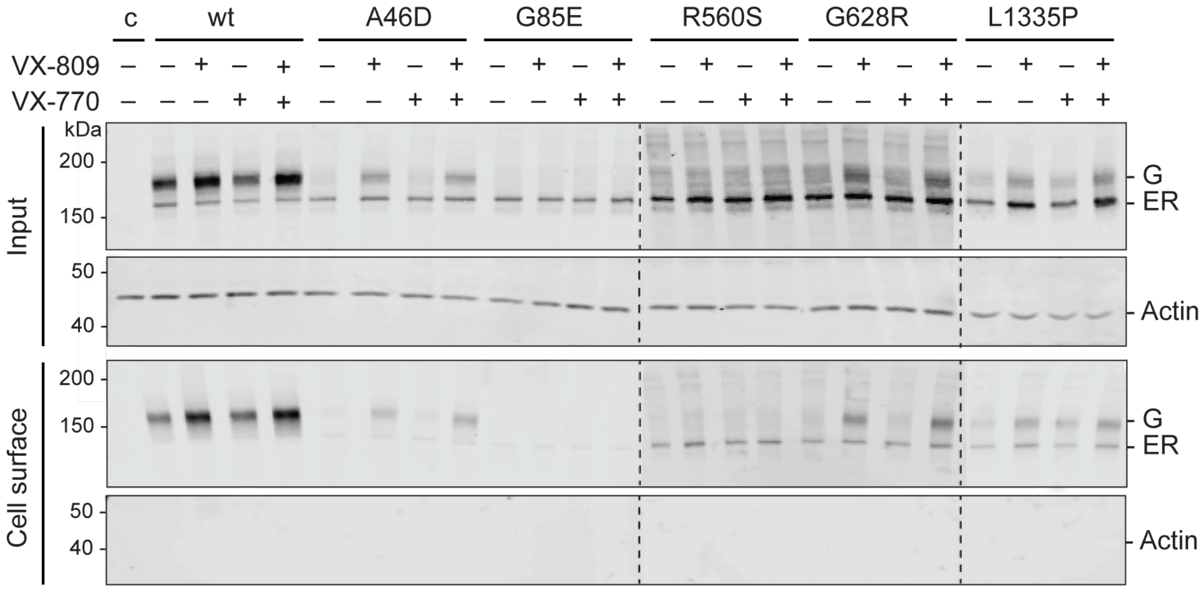

Supplementary Figure S2: Figure 1 with darker image for R560S and G628R lanes Figure 1B, but with decreased brightness for R560S and G628R CFTR panels.

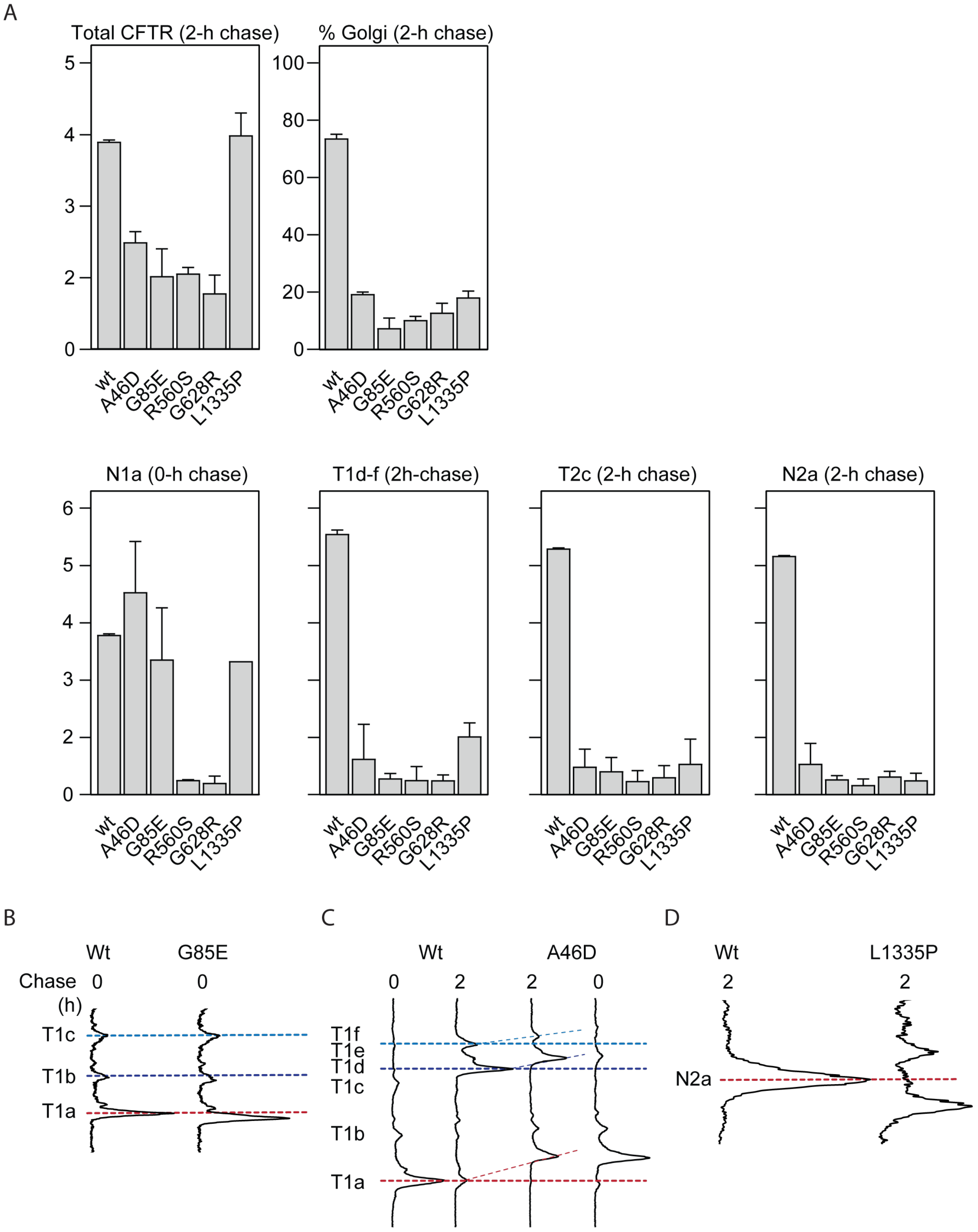

Supplementary Figure S3: Quantifications and lane profiles of hypo-responding mutants

(A) Quantification of total wild-type and mutant CFTR, percentage CFTR transported to the Golgi complex (G/(ER+G)), and CFTR fragments from experiments including Figure 2. Band intensities were quantified using ImageQuantTL and graphed as arbitrary units. Bars show standard deviations of n=3. **(B)** Lane profiles from Figure 2A show the downward shift of the

TMD1 T1a fragment in CFTR bearing the G85E mutation (red dotted line). **(C)** TMD1 fragments from A46D at 0-h and 2-h chase times deviated from the typical wild-type-like pattern. Lane profiles from Figure 4B, C without modulators show the upshift of the fragments that contain the A46D mutation. T1a does not contain A46D and disappears because L49 becomes shielded in the mutant. Light blue dotted line indicates mobility of wt T1f; dark blue dotted line indicates mobility of wt T1d, red dotted line indicates mobility of wt T1a, dark grey dotted lines represent shifts in T1f and T1d. **(D)** Lane profiles of CFTR NBD2 fragments immunoprecipitated with 596 from Figure 4C. L1335P yields a smaller NBD2 fragment than wild-type N2a, which is marked with a red dotted line.

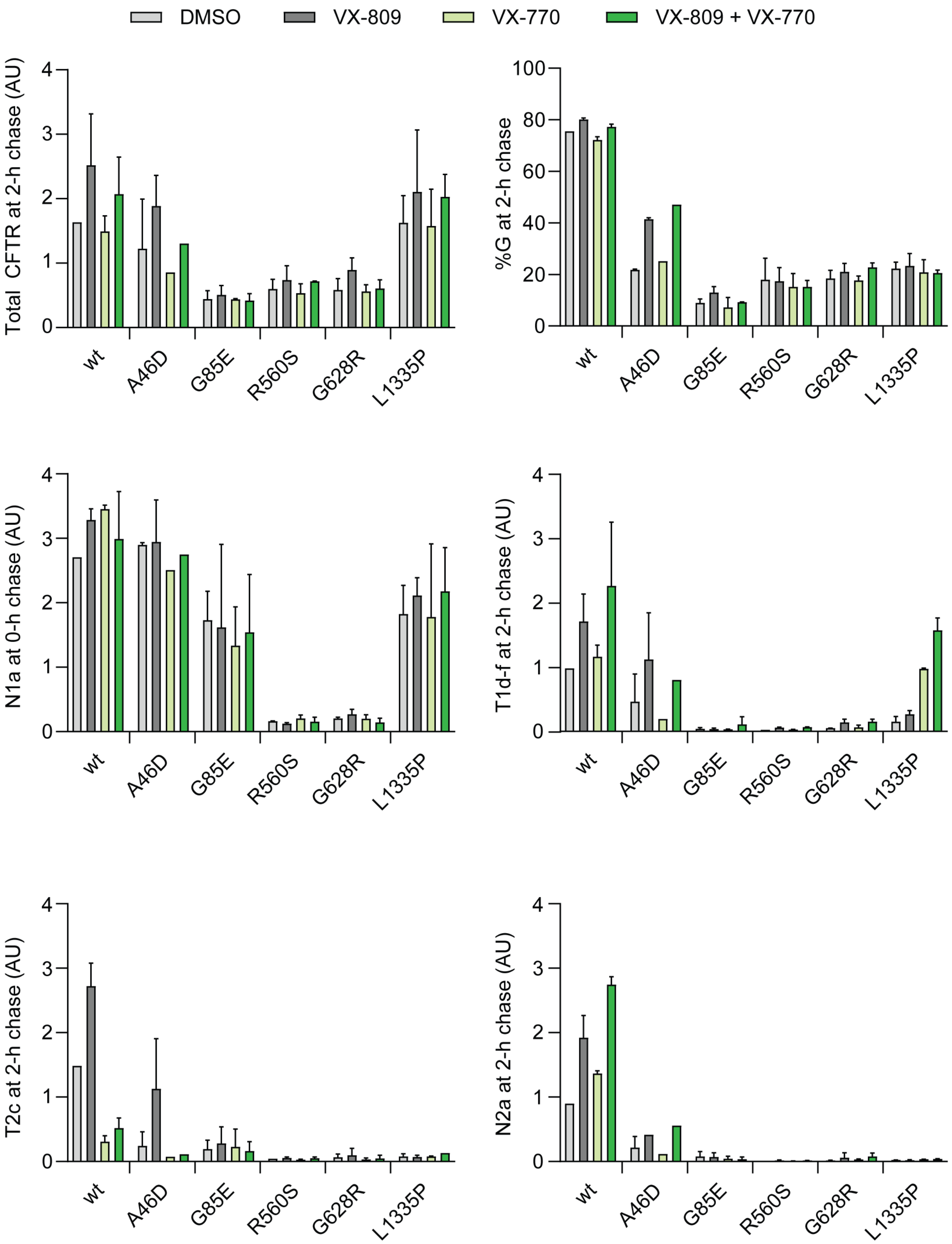

Supplementary Figure S4: Quantifications of CFTR in hypo-responding CFTR mutants Quantification of total wild-type and mutant CFTR (AU), percentage CFTR that left the Golgi compartment, and CFTR fragments (AU) as in Figure 3A.

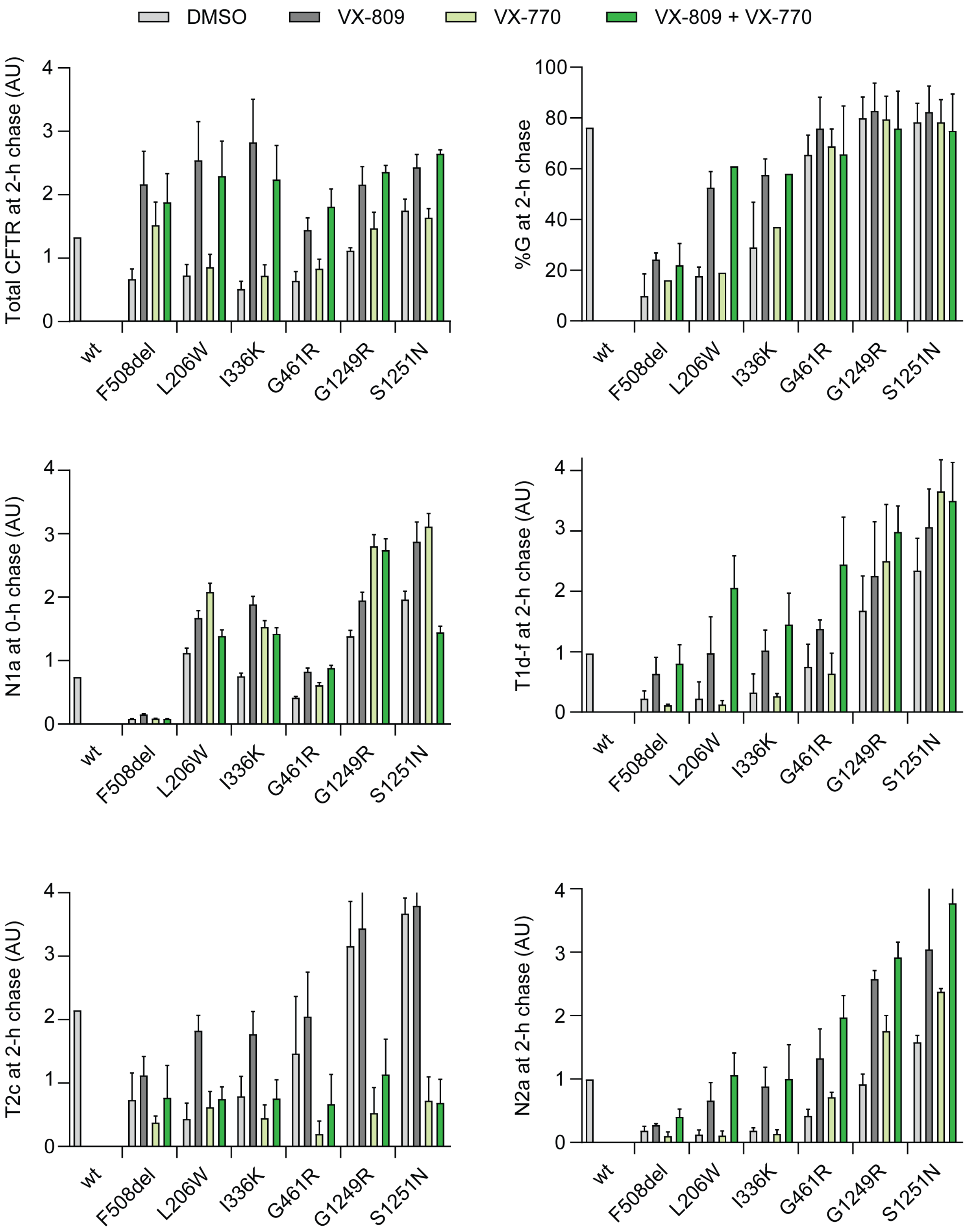

Supplementary Figure S5: Quantifications of CFTR in hyper-responding CFTR mutants Quantification of total wild-type and mutant CFTR (AU), percentage CFTR that left the Golgi compartment, and CFTR fragments (AU) as in Figure 3A.

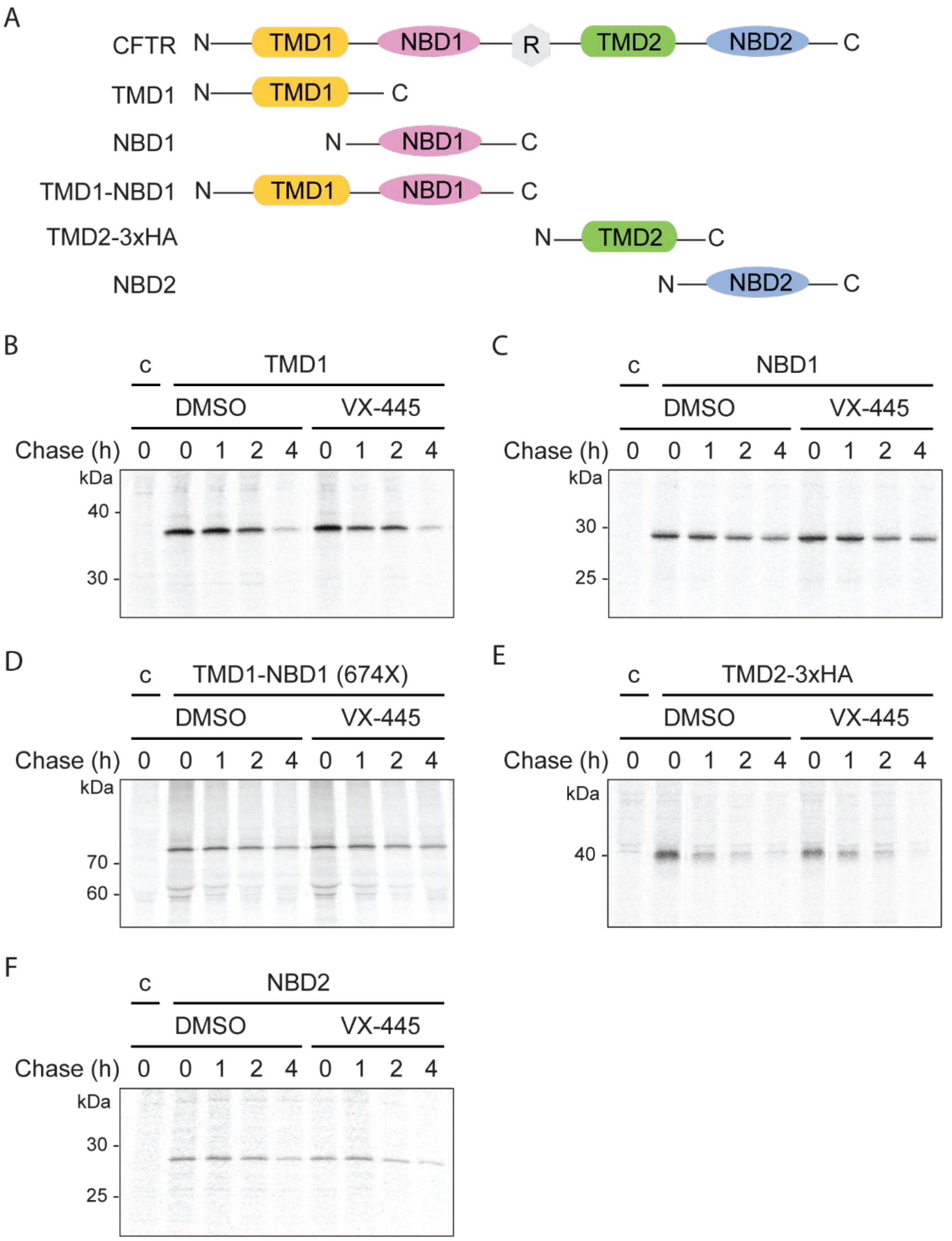

Supplementary Figure S6: Single and bi-domain CFTR constructs are not stabilized by VX-445 (quantifications in Figure S7)

(A) Schematic representation of constructs used in (B-F). **(B)** HEK293T cells expressing isolated TMD1 were analyzed as in Figure 8: radiolabeled for 5 minutes and chased for 0, 1, 2, or 4 hours. VX-445 (3 μM) was added during starvation, pulse, and chase. Cycloheximide (1 mM) was added during the chase only. CFTR was immunoprecipitated from detergent cell lysates using E1-22 and analyzed on 12% SDS-PA gels. **(C-F)** As in (B), intracellular stability of **(C)** Wild-type isolated NBD1, immunoprecipitated with MrPink, **(D)** TMD1-NBD1 (674X), immunoprecipitated with MrPink, **(E)** Isolated TMD2-3xHA CFTR, immunoprecipitated with

TMD2C, and **(F)** Isolated NBD2, immunoprecipitated with 596. Gels are representative of three repeats.

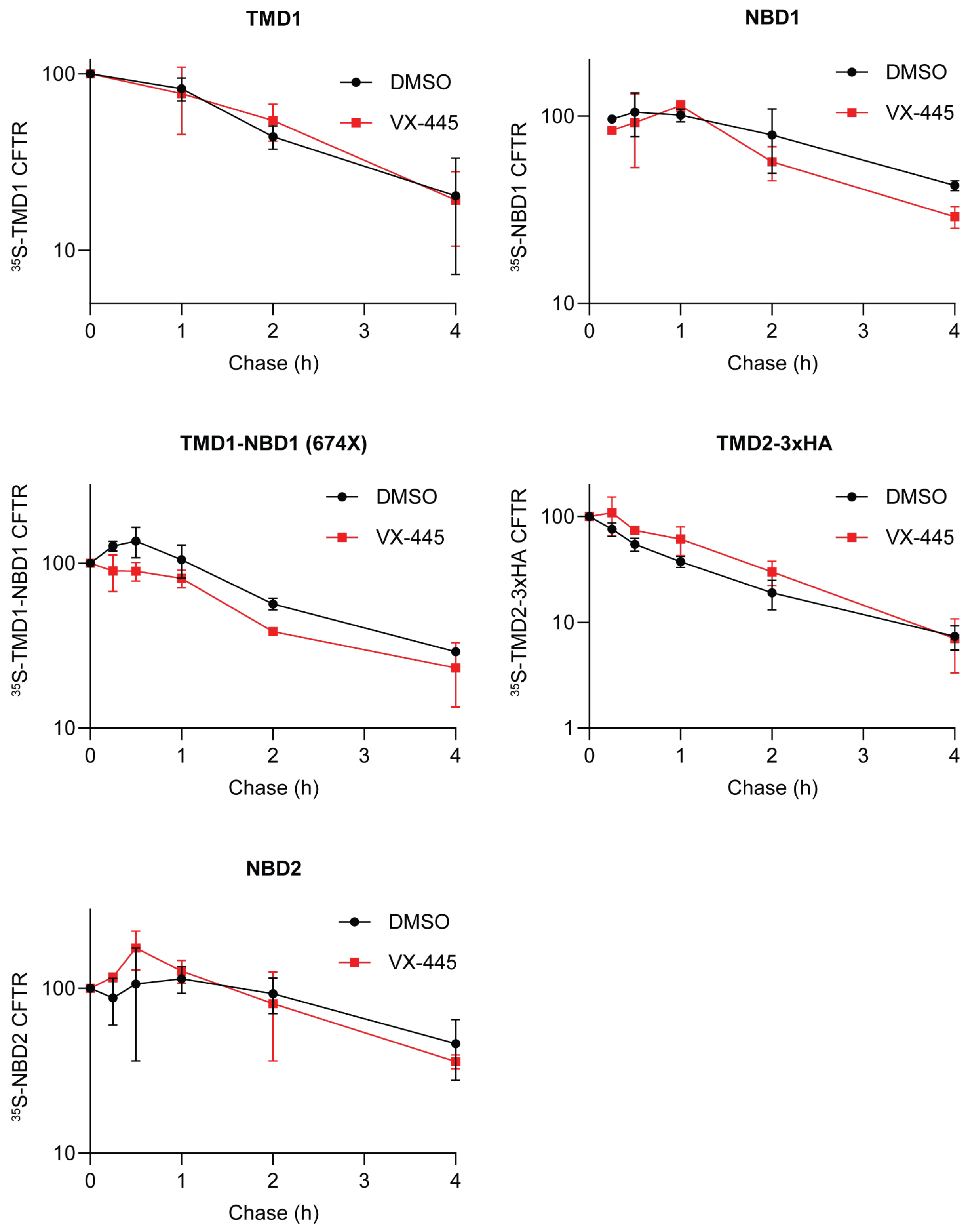

Supplementary Figure S7: Degradation curves of VX-445 treated CFTR constructs (gels in Figure S6)

Intracellular stability of single CFTR domains (TMD1, NBD1, TMD2, and NBD2) and bi-domain CFTR (TMD1-NBD1) was probed by a chase of ^{35}S-labeled protein. Total amount of CFTR that was left after 1, 2, or 4 h of chase was normalized to the 0-h chase condition. Error bars show standard deviation from three independent experiments. Intracellular stability

of CFTR in the presence of VX-445 is indicated in red. Band intensities were quantified at each time point and plotted.

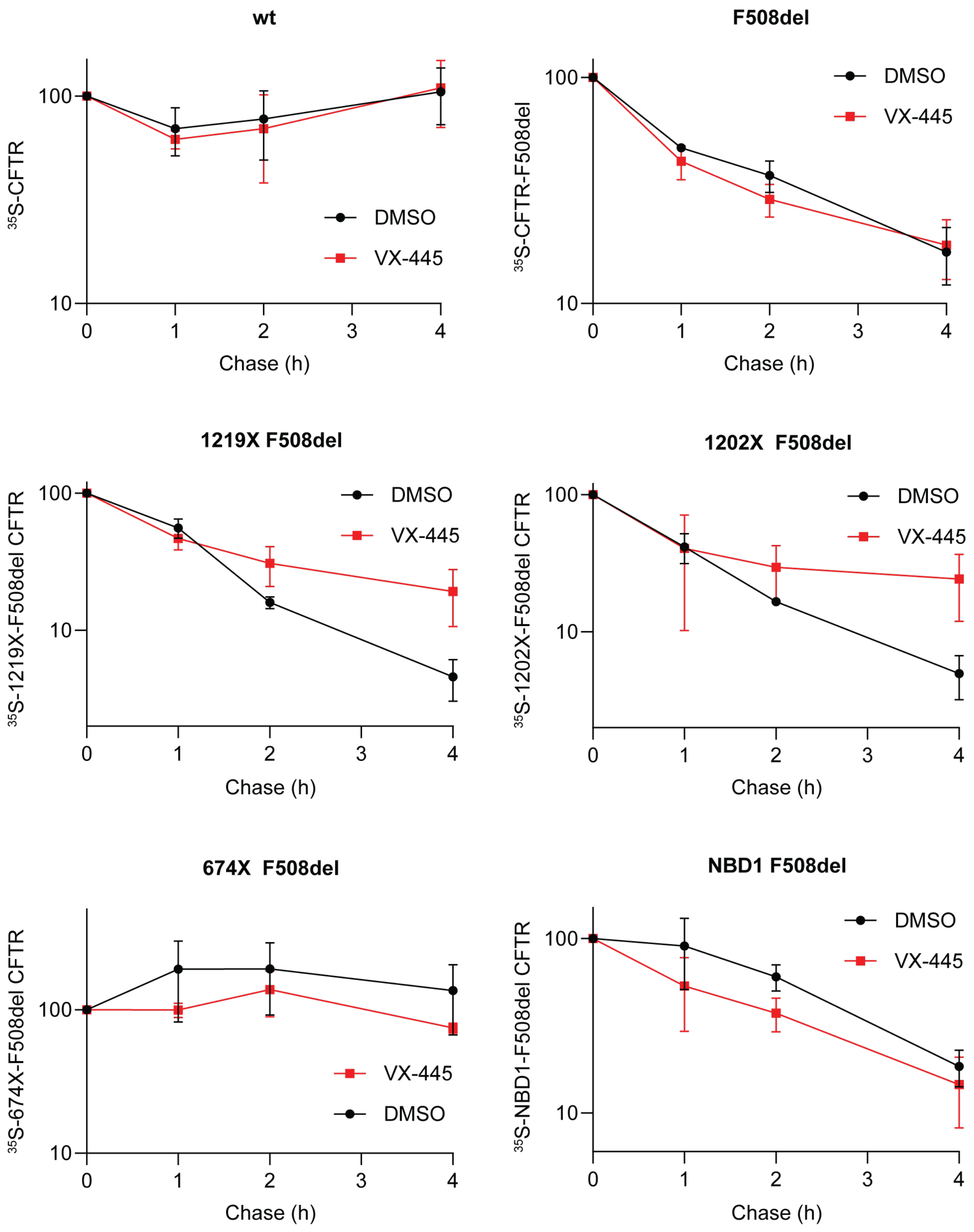

Supplementary Figure S8: Degradation curves of F508del CFTR constructs treated with VX-445 (gels in Figure 8)

Degradation curves of wild-type and F508del CFTR, and CFTR truncation constructs containing the F508del mutation (1219X-F508del, 1202X-F508del, 674X-F508del, and NBD1-F508del) were analyzed as in Figure S7. Band intensities were quantified as in Figure S7.

Table S1. Primers used for cloning of the indicated CFTR constructs

F, forward primer; R, reverse primer.

CFTR construct	Primer sequence 5' to 3'
A46D	F: GTTGATTCTGATGACAATCTATCTGAA
	R: TTCAGATAGATTGTCATCAGAATCAAC
G461R	F: TCCACTAGAGCAGGCAAGA
	R: TCTTGCCTGCTCTAGTGGA
F508del	F: CCATTAAAGAAAATATCATTGGTGTTTCCTATGATGAATATAG
	R: CTATATTCATCATAGGAAACACCAATGATATTTTCTTTAATGG
R560S	F: TCTTTAGCAAGCGCAGTATACAAAGATG
	R: CATCTTTGTATACTGCGCTTGCTAAAGA
G628R	F: AGCAGCTATTTTTATAGGACATTTTCAG
	R: CTGAAAATGTCCTATAAAAATAGCTGCT
G1249R	F: ACTGGATCAAGGAAGAGTACTTTG
	R: CAAAGTACTCTTCCTTGATCCAGT
L1335P	F: TTTCCTGGGAAGCCTGACTTTGT
	R: ACAAAGTCAGGCTTCCCAGGAAA
NBD2-CFTR (1202-1480)	F: GGGGCGGCCGCGACCATGGACATCTGGCCCTCAGGGGGCCAAATG
	R: CCTCTGGAGATATCGTCGACAAGCTTATCGATGCG
